# Sexual dysfunction therapeutic approaches in patients with multiple sclerosis: a systematic review

**DOI:** 10.1007/s10072-022-06572-0

**Published:** 2022-12-31

**Authors:** Vasileios Giannopapas, Dimitrios Kitsos, Anthi Tsogka, John S. Tzartos, Georgios Paraskevas, Georgios Tsivgoulis, Konstantinos Voumvourakis, Sotirios Giannopoulos, Daphne Bakalidou

**Affiliations:** 1 Laboratory of Neuromuscular & Cardiovascular Study of Motion (LANECASM), University of West Attica, Athens, Greece; 2 Second Dept of Neurology, Attikon University Hospital, National & Kapodistrian University of Athens, Rimini 1, 12462 Athens, Greece; 3 Department of Physiotherapy, University of West Attica, Athens, Greece

**Keywords:** Multiple sclerosis, Sexual function, Sexual dysfunction, Erectile dysfunction

## Abstract

**Objective:**

Multiple sclerosis (MS) is the most common chronic inflammatory demyelinating disease of the central nervous system (CNS). The most common clinical manifestations of MS are spasticity, pain, vesico-urethral disorders, cognitive impairments, chronic fatigue and sexual dysfunction. This review aims to explore the possible therapeutic options for managing sexual dysfunction in people with MS (PwMS).

**Method:**

A thorough search of the PubMed Medline database was performed. Records were limited to clinical studies published between 01/01/2010 up to 01/01/2022. The results were screened by the authors in pairs.

**Results:**

The search identified 36 records. After screening, 9 records met the inclusion–exclusion criteria and were assessed. The pharmacological approaches investigated the effectiveness of sildenafil, tadalafil and onabotulinumtoxinA. Of the interventional studies the non-pharmacological investigated, the effectiveness of aquatic exercises, the application of pelvic floor exercises,the combination of pelvic floor exercises and mindfulness technique, the combination of pelvic floor exercises and electro muscular stimulation with electromyograph biofeedback, the application of yoga techniques and the efficacy of assistive devices like the clitoral vacuum suction device and the vibration device.

**Conclusion:**

The management of sexual dysfunction in PwMS needs to be further investigated. A team of healthcare professionals should be involved in the management of SD in order to address not only the primary (MS-related) SD symptoms but the secondary and tertiary as well. The main limitations that were identified in the existing literature were related to MS disease features, sample characteristics and evaluation tools and batteries.

## Introduction

Multiple sclerosis (MS) is one of the most common demyelinating neurodegenerative diseases of the central nervous system (CNS), affecting approximately 2.8 million people worldwide [1]. Clinical presentation involves a vast spectrum of phenotypes where neuroinflammatory and degenerative pathophysiologic mechanisms play major roles. With time many patients experience spasticity, sensory deficits, diplopia, bladder and bowel disorders, fatigue and quite often sexual dysfunction (SD) symptoms as MS is associated with significant long‐term urogenital impairment. It has been estimated that 50–90% of men with MS and 40–80% of women with MS will experience some form of sexual dysfunction related to MS in their life [2]. According to a recent study by Schairer et al. in 2014 [3], sexual dysfunction can potentially have a more significant impact on the mental health and the quality of life (QoL), compared to the sum of neurological and functional deficits of the patients. Primary, as a consequence of lesions affecting the neural pathways involved in the regulation of sexual functions and secondary SD, attributable to different neurological disabilities both are important contributors to a significant deterioration of the patient’s QoL [4]. In addition, SD is related to mood disorders, diminished self‐esteem, impaired body image and also to psychological aspects of the relationship with the partner. SD, which is ignored in many cultures equally in women and men for different reasons, is an issue of vital importance for young patients suffering from MS [5, 6]. The literature shows that in both genders MS leads to declination of sexual desire, orgasmic dysfunction, loss of libido, diminished vaginal lubrication and spasticity during sexual activity [7]. One of the commonest barriers health care professionals face when attempting to address SD is the lack of awareness about the different pharmacological and non-pharmacological strategies available for managing sexual problems in both women and men as well as the stigma that sometimes surrounds these kind of health problems.

## Methods

**Fig. 1:**
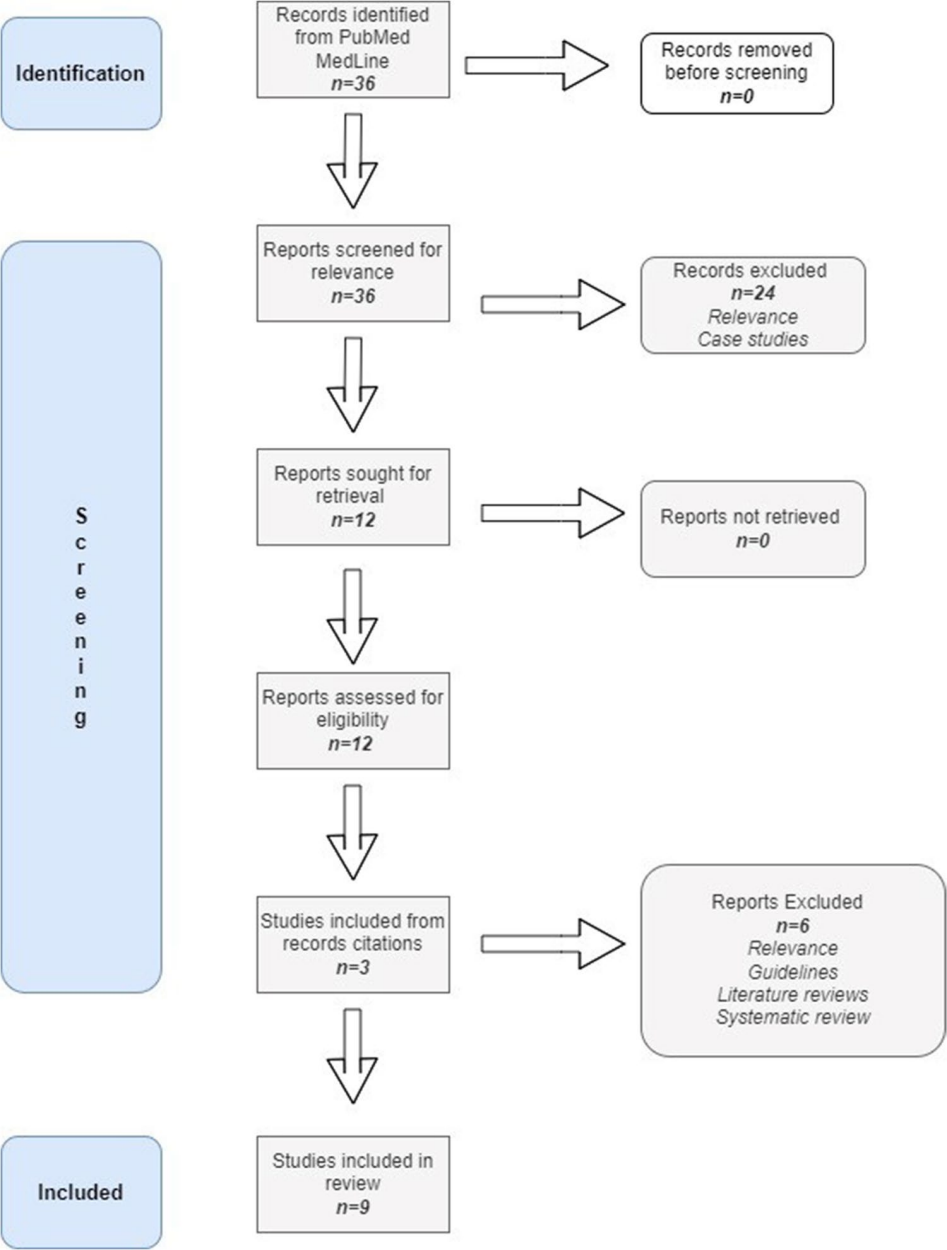
PRISMA diagram

We conducted a review of clinical studies reporting the interventional and non-interventional therapeutic approaches in PwMS with SD symptoms and an informal quality analysis of their design based on the Marrie and Wolfson criteria [8]. The authors independently performed the literature search, study selection and data extraction. Studies reporting interventional and non-interventional means of approaching SD in MS were included while protocols presenting purely surgical approaches were excluded from the literature search. The studies included were not based on a specific symptom approach but on the thorough investigation of the therapeutic approach for the symptom. Most included based the design of the study and the stratification of the MS participants with SD on the Female Sexual Function Index—FSFI or the International Index of Erectile Function-IIEF15 tools, hence the database search was adjusted accordingly. Pubmed MEDLINE database was accessed covering the period from 01/01/2010 up to 01/01/2022 using the following search terms: “(“Multiple Sclerosis”[Mesh]) AND (“Sexual Dysfunction, Physiological/drug effects”[Mesh] OR “Sexual Dysfunction, Physiological/drug therapy”[Mesh] OR “Sexual Dysfunction, Physiological/prevention and control”[Mesh] OR “Sexual Dysfunction, Physiological/rehabilitation”[Mesh] OR “Sexual Dysfunction, Physiological/surgery”[Mesh] OR “Sexual Dysfunction, Physiological/therapy”[Mesh])”. Retrieved studies from the initial search were further screened for additional articles. The studies presenting the above mentioned data are presented in Table 1. The corresponding flow chart is presented in Fig. 1.

**Table 1:** Studies included

*Author–date*	*Population*	*MS type*	*Duration*	*Intervention*	*Evaluation*	*Results*	*Study Design Rating*
*Saferinejad *et al*. (2009)*	Male PwMS *N* = 203	> 1 relapse 1 year pre-entry	24 attempts	Sildenafil 50–100 mg Group A: *n* = 102 (active) Group B: *n* = 101 (placebo)	IIEF-q3, q4 SEP2-3 GAQ	Sildenafil does not constitute a “routine” intervention for erectile dysfunction in MS	A
*Najafidoulatabad *et al*. (2014)*	Female PwMS *N* = 60	n/a	3 months	Yoga and exercise *Group A*: *n* = 30 (case group) *Group B*: *n* = 30 (control)	MSQoL-54	Significant statistical differences between the 2 groups regarding physical ability and sexual function-satisfaction (*p* = .001)	B
*Lucio *et al*. (2014)*	Female PwMs *N* = 30	RRMS	12 weeks	*Group A*: PMFT, EMG biofeedback, sham NMES *Group B*: PMFT, EMG biofeedback, intravaginal NMES *Group C*: PMFT, EMG biofeedback, TTNS	PERFECT scheme FSFI	Group B Improved PFM tone (*p* = .01) Decrease in vaginal flexibility (*p* = .02)	A
*Gianantonni *et al*. (2015)*	Female PwMS *N* = 41	4 SPMS 21 RRMS	3 months	*Onabot/A intravesical intradetrusorial injection* *Active*: 41 *Control*: 21	I-QoL FSFI HAM-A/HAM-D 3-day void diary	Improvement in urination (decrease in 3-day diary) (*p* < .001) Increased FSFI (desire *p* < .005, arousal *p* < .0005, lubrication *p* < .005, orgasm *p* < .005, satisfaction *p* < .004)	A
*Francomano *et al*. (2016)*	Male PwMs *N* = 30	n/a	4 weeks	Tadalafil 5 mg/daily *Group A*: *n* = 20 (active) *Group B*: *n* = 10 (placebo)	IPSS IIEF-15	*Group A* Improved IPSS score (*p* < .001) Improved IIEF-5 score (*p* < .001)	*B*
*Alexander *et al*. (2017)*	Female PwMs *N* = 20 Female PwSCI *N* = 11	n/a	12 weeks	*Clitoral vacuum suction device* *(CVSD group)* Nms = 13 Nsci = 16 *Vibration stimulation* *(VS group)* Nms = 10 Nsci = 15	FSFI FSDS	CVSD group Improved FSFI (*p* = .11) Decreased FSDS (up to 4 weeks after treatment) VS group Improved orgasm FSFI *(p* = .28) subscale	C
*Mosalanejad *et al*. (2018)*	Female PwMs *N* = 70	In Remission	8 weeks	Pelvic floor exercise -Mindfulness Group A: PFE Group B: mindfulness Group C: combination	FSFI	Improved FSFI No significant difference in sexual function between Groups A, B and C (*p* > .05)	C
*Bahmani *et al*. (2020)*	Female PwMS *N* = 60	n/a	8 weeks	A: aquatic exc. × 2/week B: aquatic exc. × 3/week C: active control condition	FSFI Couple satisfaction BDI-FS FSS	Improved sexual function scores (FSFI) groups A, B vs C *p* < .001 Desire *p* = .002, arousal *p* = 0.1, lubrication *p* = .11, orgasm *p* = .007, satisfaction *p* = .23, pain *p* = .2	A
*Altunan *et al*. (2021)*	Male PwMS *N* = 9 Female PwMS *N* = 33	RRMS	12 weeks	Pelvic floor exercise program *Group A*: LUTS + *Group B*: LUTS −	ICIQ-SF BDI MSQoL-54 FSFI/SHIM MBV, PVR (ultrasonography)	Increased MBV (*p* < .01) Increased FSFI (*p* = .02) Decreased BDI (*p* > .05) Decreased ICIQ-SF (*p* > .05) Reduced depression (*p* > .05)	B

*BDI* Beck Depression Inventory, ΕHS Erection Hardness Scale, EMG electromyography, *FSDS* Female Sexual Distress Scale, *FSFI* Female Sexual Function Index, *FSS* Fatigue Severity Scale, *GAQ* global assessment question, *HAM-A* Hamilton Anxiety Rating Scale, *HAM-B* Hamilton Depression Rating Scale, *ICIQ-SF* International Consultation on Incontinence Questionnaire-Urinary Incontinence Short Form, *IIEF q2–q3* sexual function and sexual satisfaction, *IIEF-15* International Index of Erectile Function, *IPSS* International Prostate Symptom Score, *LSC* Life Satisfaction Questionnaire (sexual life, family life and partner life questions), *LUTS* lower urinary tract symptoms, MBV Max Bladder Volume, MS Multiple Sclerosis,MSQolL-54 Multiple sclerosis Quality of Life Questionnaire, *PFMT* pelvic floor muscle exercises, *PVR* post-void residual, QoL quality of life, *SEP2–3* sexual encounter profile questions 2, 3 (penetration and duration of erection), *SHIM* Sexual Health Inventory of Men, *SCI* Spinal cord injury, *TTNS* transcutaneous tibial nerve stimulation

## Results

There was a total of 9 [8–16] trials about the treatment approaches on SD. Two of them were pharmacological approaches of which one was grade A; one was grade B [9, 10]; one was interventional and graded with A [11]; and 6 non-pharmacological and non-interventional protocols which were graded as follows: 2 studies with A [12, 15], 2 with B [13, 17] and 2 with C [14, 16].

The pharmacological approaches investigated the effectiveness of sildenafil, tadalafil and onabotulinumtoxinA [9–11]. Among the interventional studies the non-pharmacological investigated, the effectiveness of aquatic exercises, the application of pelvic floor exercises, the combination of pelvic floor exercises and mindfulness technique, the combination of pelvic floor exercises and electro muscular stimulation (EMS) with electromyograph biofeedback (EMG-BF), the application of yoga techniques and the efficacy of adaptive devices (AD) like the clitoral vacuum suction device (CVSD) and the vibration device (VD) [12–17].

### Pharmacological studies

A study by Safarinejad and colleagues [9] examined the safety and efficacy of sildenafil citrate in the management of erectile dysfunction in 100 male patients with a verified diagnosis of MS (mean age 41 years, mean disease duration 11.3 years and mean EDSS score 2.7) with at least 1 clinical relapse during the last year versus 100 male patients with verified diagnosis (mean age 40 years, mean disease duration 11.7 years and mean EDSS score 2.8). The researchers concluded that sildenafil citrate versus placebo had little effect on erectile dysfunction due to multiple sclerosis and therefore cannot be recommended as routine treatment. On the other hand, Francomano and colleagues [10] concluded that a daily 5-mg regimen of tadalafil improved erectile function in the active MS group (*p* < 0.001), versus the non-active MS control group and had similar beneficial effects on the urine bladder function (mean age 44.5 years, mean disease duration 14.5 years and no EDSS data provided—data given for the whole of the participants). In the only interventional pharmacological study, Gianantonni and colleagues [11] concluded that in 41 female MS patients with a confirmed diagnosis (mean age 44.5 years, disease duration 16.2 and mean EDSS score 4.5) versus 21 control clinically definite MS cases (mean age 43.2 years, disease duration 15.2 and mean EDSS score 3.4) the administration of onabotulinum toxin A (OnaBot/A) via intra detrusorial injections improved sexual function, 3 months after the injection, in approximately 70% of the continent patients (desire *p* < 0.005, arousal *p* < 0.0005, lubrication *p* < 0.005, orgasm *p* < 0.005 and satisfaction *p* < 0.004). The beneficial effects of OnaBot/A in sexual function seems to be a combination of the positive impact on the urological and psychological patient status.

### Non-pharmacological studies

#### Aquatic exercise protocol

Bahmani and colleagues [12] conducted a study which included 60 female MS patients who were randomly assigned in three groups: aquatic exercise 2 times a week (mean age 39.3 years, disease duration 8.8 and mean EDSS score 3.0), aquatic exercise 3 times a week (mean age 40.6 years, disease duration 6.5, mean EDSS score 1.5) and an active control conditioning (non-specific aquatic and non-aquatic exercises—mean age 33.7 years, disease duration 6.3 and mean EDSS score 1.5) for 8 weeks. The interventions lasted 8 weeks and were monitored using questionnaires regarding their sexual function (FSFI), symptoms of depression (BDI-FS), sleep complaints (Insomnia Severity Index), fatigue (FSS) and couple satisfaction before and after. Sexual function (*p* < 0.001) regarding (desire *p* = 0,002, arousal *p* = 0.1, lubrication *p* = 011, orgasm *p* = 0.007, satisfaction *p* = 0.23 and pain *p* = 0.2) were higher in the interventional group versus the active control group. In addition, the 2/week exercise frequency was found more beneficial, although it did not reach statistical significance.

#### Pelvic floor exercises

A study by Altunan and colleagues [13] in 42 relapse remit multiple sclerosis (RRMS) patients (mean age 43.6 years, disease duration 9.2 years and mean EDSS score 2.3–78% female patients) concluded that the effect of pelvic floor exercise program (PFEP) in the management of SD was beneficial in terms of a significant improvement in the desire and orgasm parameters of the female patients (*p* = 0.2). The effect of the PFEP was evaluated using the ICIQ-SF (bladder symptoms), the Beck Depression Index (BDI) (depression symptoms), the Multiple Sclerosis Quality of Life-54 Questionnaire (MSQoL-54) (quality of life), the FSFI (Female Sexual Function Index-for women) or the SHIM (Sexual Health Inventory for Men) to evaluate sexual health. On the other hand there was no significant effect on the sexual function of the male patients post-PFEP protocol administration.

#### Pelvic floor exercises and mindfulness

Mosalanejad and colleagues [14] concluded that there was no significant impact on the sexual function from the combination of PFE and mindfulness for 12 weeks in a three-arm parallel study on 70 female MS patients in remission period. The three groups (group A: pelvic floor muscle exercise—mean age 36 years, disease duration 59.4 months and no EDSS data given; group B: mindfulness, group—mean age 35.7 years, disease duration 53.2 months and no EDSS data given; group C: combination of pelvic floor exercise and mindfulness—mean age 35.5 years, disease duration 63.5 months and no EDSS data given) were asked to perform either pelvic muscle and/or mindfulness for 12 weeks.

#### Pelvic floor exercises and EMS

Lucio and colleagues [15] aimed to assess the effectiveness of pelvic floor muscle training (PMFT) alone or with EMS in SD in 30 female RRMS patients. The participants were divided in 3 equal groups (group 1: mean age 44.5 years, disease duration 15 years and EDSS score 3.5; group 2: mean age 47 years, disease duration 12 years and EDSS score 4.5; group 3 mean age 47 years, disease duration 11 years and EDSS score 4.0) were PFMT and EMS with electromyograph biofeedback (EMG-BF) were applied via different administration protocols. The researchers concluded that PFE alone or in combination with EMS in the form of neuromuscular electric stimulation (NMES) or transcutaneous tibial nerve stimulation (TTNS) improves sexual function in MS (*p* = 0.01).

#### Adaptive devices (CVSD-VD)

Alexander and colleagues [16] conducted a mixed population randomized control trial (RCT) which consisted of 31 female participants, 20 MS (mean age 46.7 years, disease duration 13.7 years and no EDSS data given) and 11 with spinal cord injury (SCI) in order to examine the safety and efficacy of CVSD versus vibratory stimulation, via a VD, in orgasmic dysfunction. The main premise of the study lay in the association of sacral reflexes with the ability to achieve orgasm and the main hypothesis was that the retraining of sacral responses through either CVSD or VS would help in treating female orgasmic dysfunction. The study lasted 12 weeks and the participants were trained on how to use either device and given instructions to use it any time (either alone, prior, or during sexual activity). The authors concluded that the CVSD was more beneficial for orgasmic and overall sexual function in contrast to the VD, which improved orgasm but not overall sexual function for up to 4 weeks after the intervention (*p* = 0.11).

#### Application of yoga technique

Najafidoulatabad and colleagues [17] conducted an RCT to assess the effects of yoga in sexual function in 60 female patients with clinically definite MS (mean age 31.6 years and no other demographic data provided). Women in the case group were asked to perform a series of yoga training and exercises for 3 months (8 sessions per month). Pre- and post-study evaluations of the effects were measured using the MSQoL-24 Questionnaire. After the 3-month mark, there was a significant statistical difference in sexual satisfaction in the case group participants (*p* = 0.001), while the women in the control group showed exacerbated symptoms.

## Discussion

SD is a common problem that can lead to the deterioration of both physical and mental health and can have a greater impact on the QoL, compared to the level of physical disability [3] but despite its high prevalence and impact on QoL, interventional studies in PwMS are very few. Regarding especially pharmacological interventions, literature studies concluded that sildenafil had no effect on MS related SD in male patients [9] (grade A); tadalafil significantly improved erection [10] based on IIEF5 Questionnaire (grade B); and OnaBot/A improved desire, arousal, lubrication, orgasm quality and satisfaction levels, based on FSFI Questionnaire, mainly in continent female patients [11] (grade A). A number of trials explored different non-pharmacological modalities and psychoeducation in different settings over a variable number of months (usually 8–12 weeks) which improved scores regarding sexual desire and arousal, lubrication, satisfaction and pain [12–17]. PFE alone (grade B) or in combination with EMS with EMG-BF (grade A) or mindfulness techniques (grade C) [13–15], aquatic exercise protocols [12] (grade A), yoga exercise protocols [17] (grade B) and ASD [16] (grade C) applications show promise in improving different domains of SD.

After the quality evaluation of the design as well as the safety of investigated interventions of each protocol, the authors believe that a combination of physical exercises (such as yoga, pelvic floor exercises and aqua therapy) should be the first step when addressing SD in both men or women. Following that, the use of assistive devices and pharmacological treatment (specifically tadalafil 20 mg for men with SD and onabot/A intradetrusorial injection for women with SD) can further help in restoring the patient’s sexual function.

Generalization of these results to a larger cohort of PwMS is restricted by small sample sizes, the inclusion of perimenopausal women, heterogeneous levels of disability, lack of information about psychological comorbidities, medication use i.e. antidepressants and spasticity symptomatic medication, lack of long‐term follow‐up and the use self-reporting questionnaires which tend to either exaggerate, via the use of emotional language, the severity of a SD symptom, or tend to underestimate it, as it may represent some kind of stigma for the participant to mention.

Future protocols investigating SD in PwMS should focus on optimizing sample selection criteria, to promote homogeneity of MS features and control for primary, secondary and tertiary SD symptoms. Sex-specific variables such as gender, sexual orientation as well as the socio-economic status and cultural background should be accounted in the construction of any SD management related protocol (i.e. in some cases the participant may feel more comfortable talking to a male or a female health care professional (HCP) or someone with the same cultural background). In addition, non-invasive devices that may assess physiological processes like male or female erection (ultrasonic shear wave elastography for male patients [18] and transvaginal ultrasonography for female ones seem to have a more objective approach to SD evaluation [19]). Lastly, SD is a multi-dimensional condition, affected by physiological, psychological and socio-economic factors especially in PwMS [4], thus any therapeutic approach aiming in managing or restoring a patient’s sexual function must be carried out by a multi-faceted team of HCPs (neurologist, gynaecologist, urologist, physical therapist, psychiatrist, psychotherapist, social workers and more) to achieve a holistic approach with the most effective therapeutic result.

In conclusion, SD is highly prevalent in both male and female PwMS. Different demographic and MS‐specific characteristics are associated with SD, in particular advancing disease, progressive MS subtypes and presence of bladder dysfunction. The variable prevalence of SD in different studies highlights the need for a validated MS‐specific questionnaire and other more objective assessment tools, to accurately capture profile characteristics and impact on QoL. Management of SD in MS should involve a variety of HPs to properly address all possible factors that affect the patient’s sexual function and satisfaction.

## Data Availability

No data availability is needed since it is a systematic review.

## Abbreviations

AD: Assistive devices
BDI: Beck Depression Inventory
CNS: Central nervous system
CVSD: Clitoral vacuum suction device
EDSS: Expanded Disability Severity Scale
ΕHS: Erection Hardness Scale
EMG-BF: Electromyograph biofeedback
EMS: Electro muscular stimulation
FSDS: Female Sexual Distress Scale
FSFI: Female Sexual Function Index
FSS: Fatigue Severity Scale
GAQ: Global assessment question
HAM-A: Hamilton Anxiety Rating Scale
HAM-D: Hamilton Depression Rating Scale
ICIQ-SF: International Consultation of Incontinence Questionnaire—Short Form
IIEF-15: International Index of Erectile Function
IPSS: International Prostate Symptom Score
LSC: Life Satisfaction Questionnaire (sexual life, family life, partner life questions)
LUTS: Lower urinary tract symptoms
MS: Multiple sclerosis
MSFC: Multiple Sclerosis Functional Capacity
MSQoL-54: Multiple Sclerosis Quality of Life-54 Questionnaire
MBV: Max bladder volume
OnaBot/A: OnabotulinumtoxinA
PFE: Pelvic floor exercise
PFEP: Pelvic floor exercise program
PFMT: Pelvic floor muscle training
PVR: Post-void residual
PwMS: Person/people with multiple sclerosis
QoL: Quality of life
RTC: Randomized control trial
SHIM: Sexual Health Index for Men
SD: Sexual dysfunction
SEP2-3: Sexual Encounter Profile questions 2, 3 (penetration, duration of erection)
SCI: Spinal cord injury
TTNS: Transcutaneous tibial nerve stimulation
VD: Vibration device
